# Isocorroles as Homoaromatic NIR-Absorbing Chromophores: A First Quantum Chemical Study

**DOI:** 10.1038/s41598-018-29819-3

**Published:** 2018-08-10

**Authors:** Cina Foroutan-Nejad, Simon Larsen, Jeanet Conradie, Abhik Ghosh

**Affiliations:** 1 CEITEC – Central European Institute of Technology, Masaryk University, Kamenice 5, CZ – 62500 Brno, Czech Republic; 2 Department of Chemistry, UiT – The Arctic University of Norway, 9037 Tromsø, Norway; 3 Department of Chemistry, University of the Free State, 9300 Bloemfontein, Republic of South Africa

## Abstract

Density functional theory calculations of magnetically induced current densities have revealed high diatropic ring currents in unsubstituted isocorrole consistent with homoaromatic character. An examination of the Kohn-Sham molecular orbitals showed clear evidence of homoconjugative interactions in four occupied π-type molecular orbitals as well as in the LUMO. Remarkably, substituents at the saturated *meso* position were found to exert a dramatic influence on the overall current density pattern. Thus, whereas bis(trimethylsilyl)-substitution strongly enhanced the peripheral diatropic current (consistent with enhanced homoaromaticity), difluoro-substitution engendered a strong, net paratropic current (consistent with antihomoaromaticity). In this respect, isocorroles stand in sharp contrast to benzenoid aromatics, for which substituents typically exert a small influence on the current density distribution.

## Introduction

**Figure 1:**
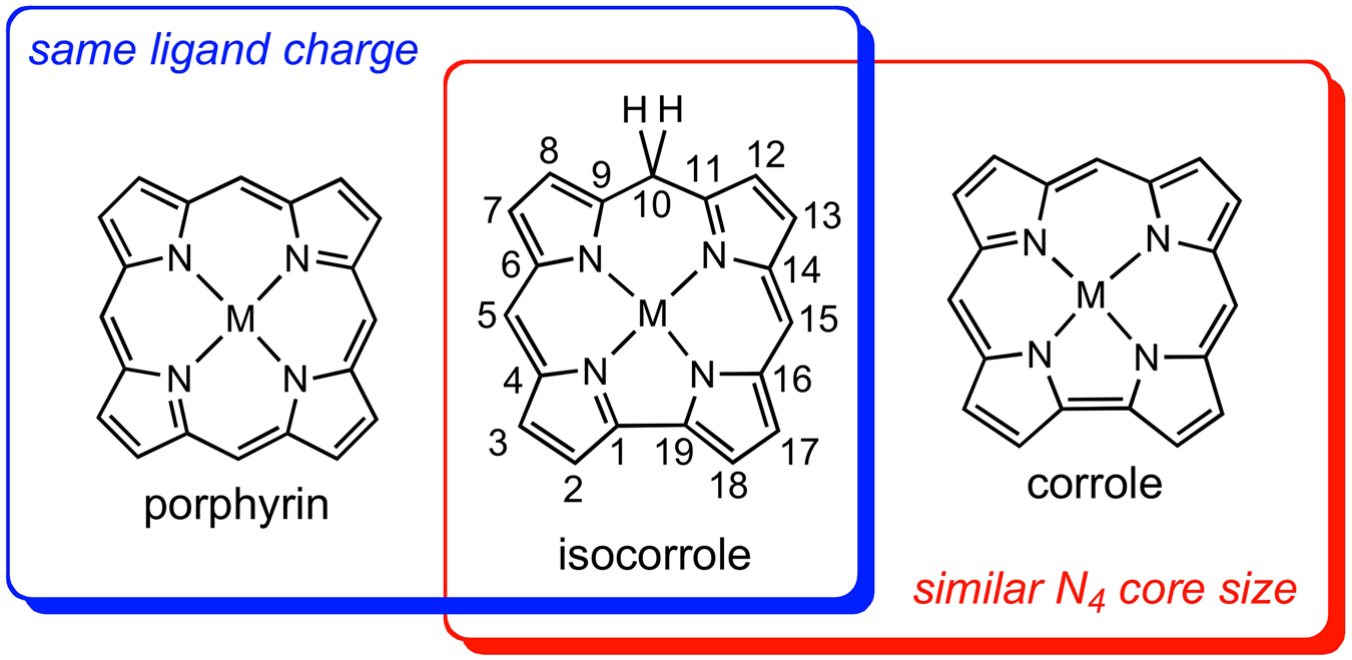
Isocorroles (with atom numbering of the carbon skeleton) as hybrid ligands with characteristics of both porphyrins and corroles.

**Figure 2:**
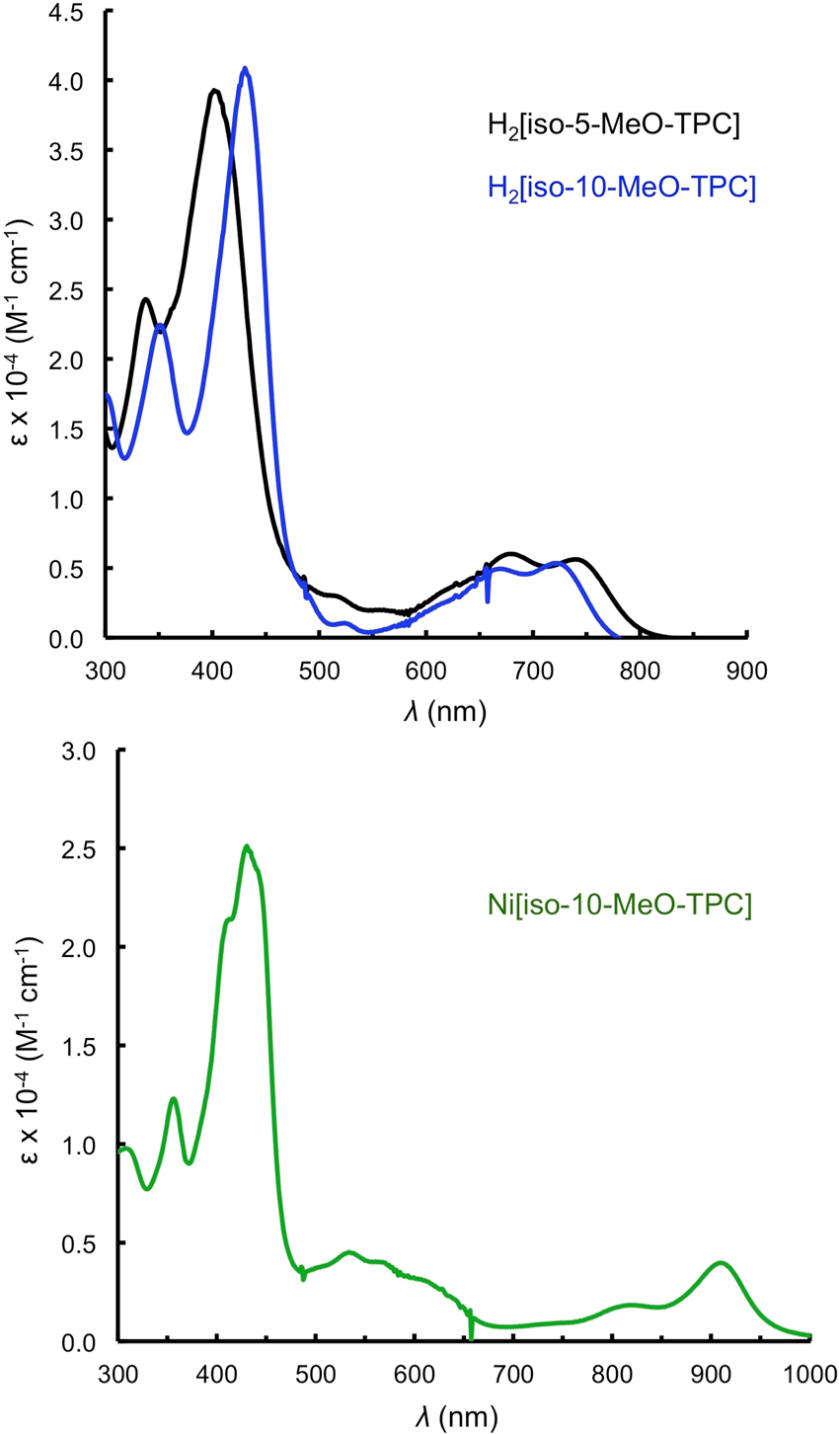
UV-vis spectra of representative isocorrole derivatives.

**Figure 3:**
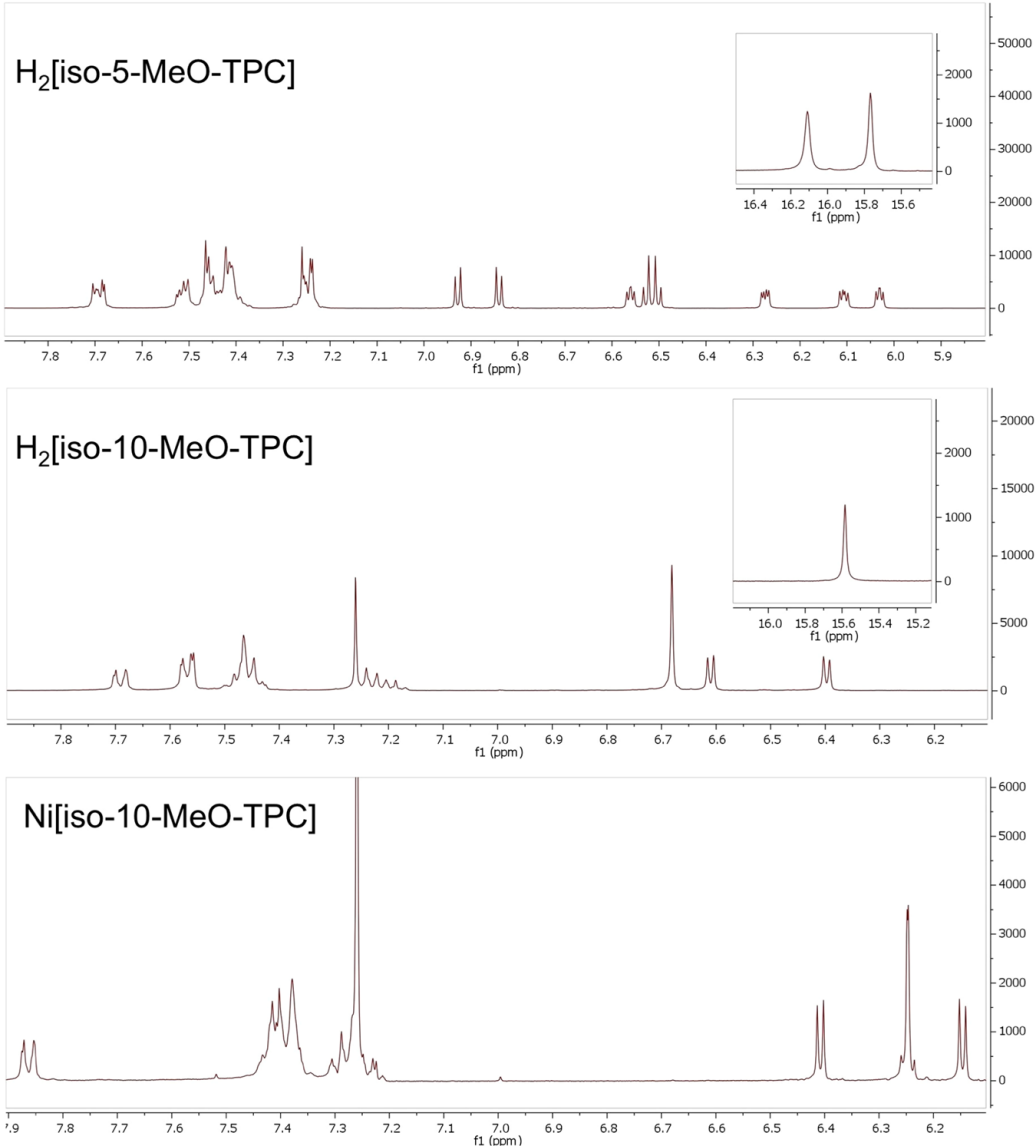
^1^H NMR spectra of representative isocorrole derivatives.

**Figure 4:**
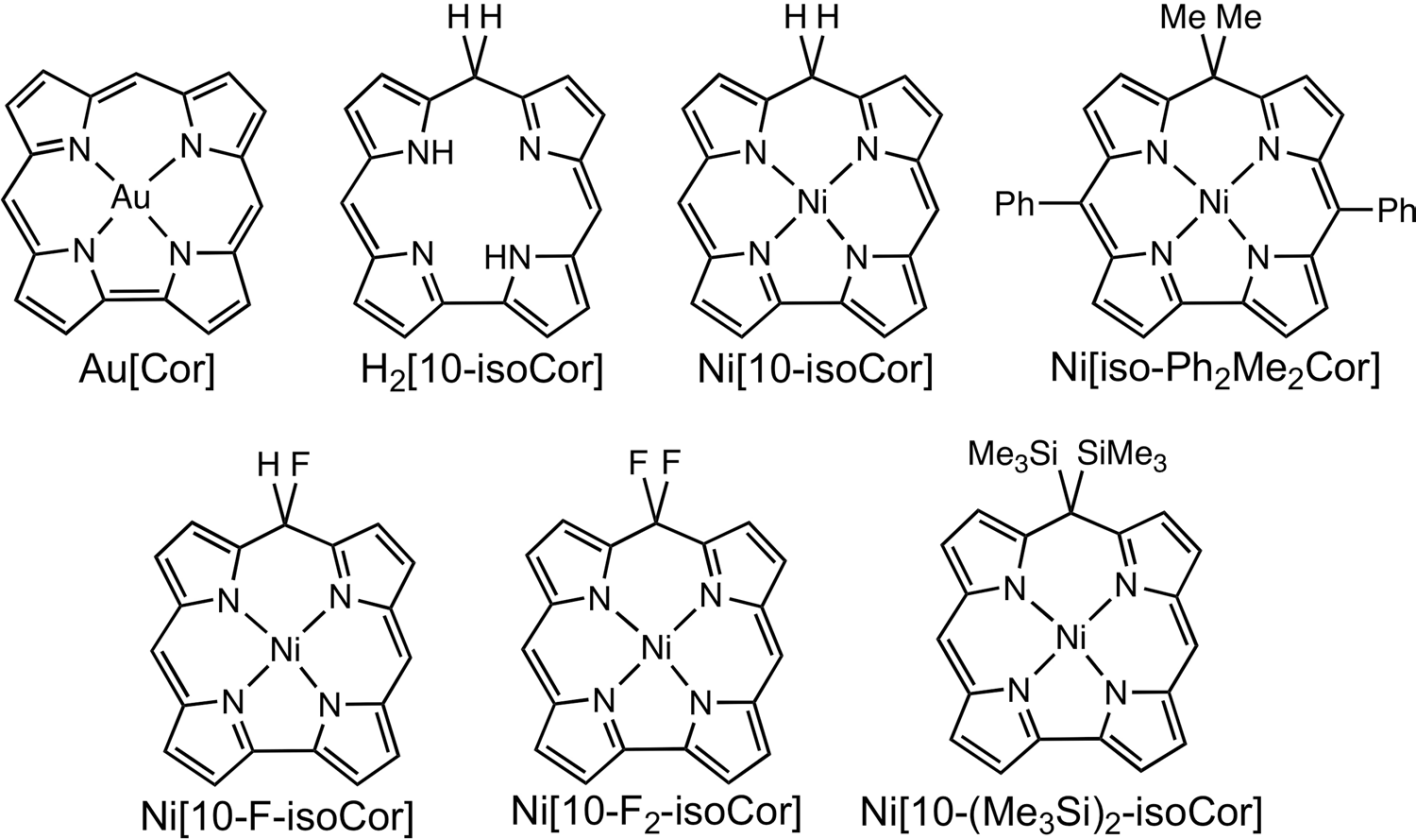
Corrole and isocorrole derivatives examined in this study.

Isocorroles are fascinating macrocyclic ligands with a sterically constrained N_4_ cavity characteristic of corroles and with the 2– charge of porphyrins (Fig. 1)^1–5^. With significant absorption in 700–1000 nm range, they are of considerable interest as near-IR dyes^6^. They also exhibit a Soret-like band in the 400–500 nm range, with an intensity comparable to those of porphyrins and corroles. These characteristics are exemplified in Fig. 2, which depicts the UV-vis spectra of selected 5/10-methoxy-5,10,15-triphenylisocorrole derivatives, H_2_[iso-5/10-MeO-TPC] and Ni[iso-5/10-MeO-TPC]. In addition, the ^1^H NMR spectra of many free-base isocorroles (including H_2_[iso-5/10-MeO-TPC]) exhibit moderately upfield-shifted *β*-pyrrole resonances and dramatically downfield-shifted NH resonances (relative to analogous corroles) (Fig. 3). These spectroscopic features are suggestive of either homoaromaticity or antihomoaromaticity, which are associated with the presence of a ring current in organic molecules in which an *sp*^3^ atom interrupts the conjugation^7–9^. Two density functional theory-based approaches have been employed here to examine the potential homoaromaticity of select isocorrole derivatives (Fig. 4), magnetically induced current density analysis and time-dependent density functional theory (TDDFT) calculations.

## Results and Discussion

### Current density analyses

**Figure 5:**
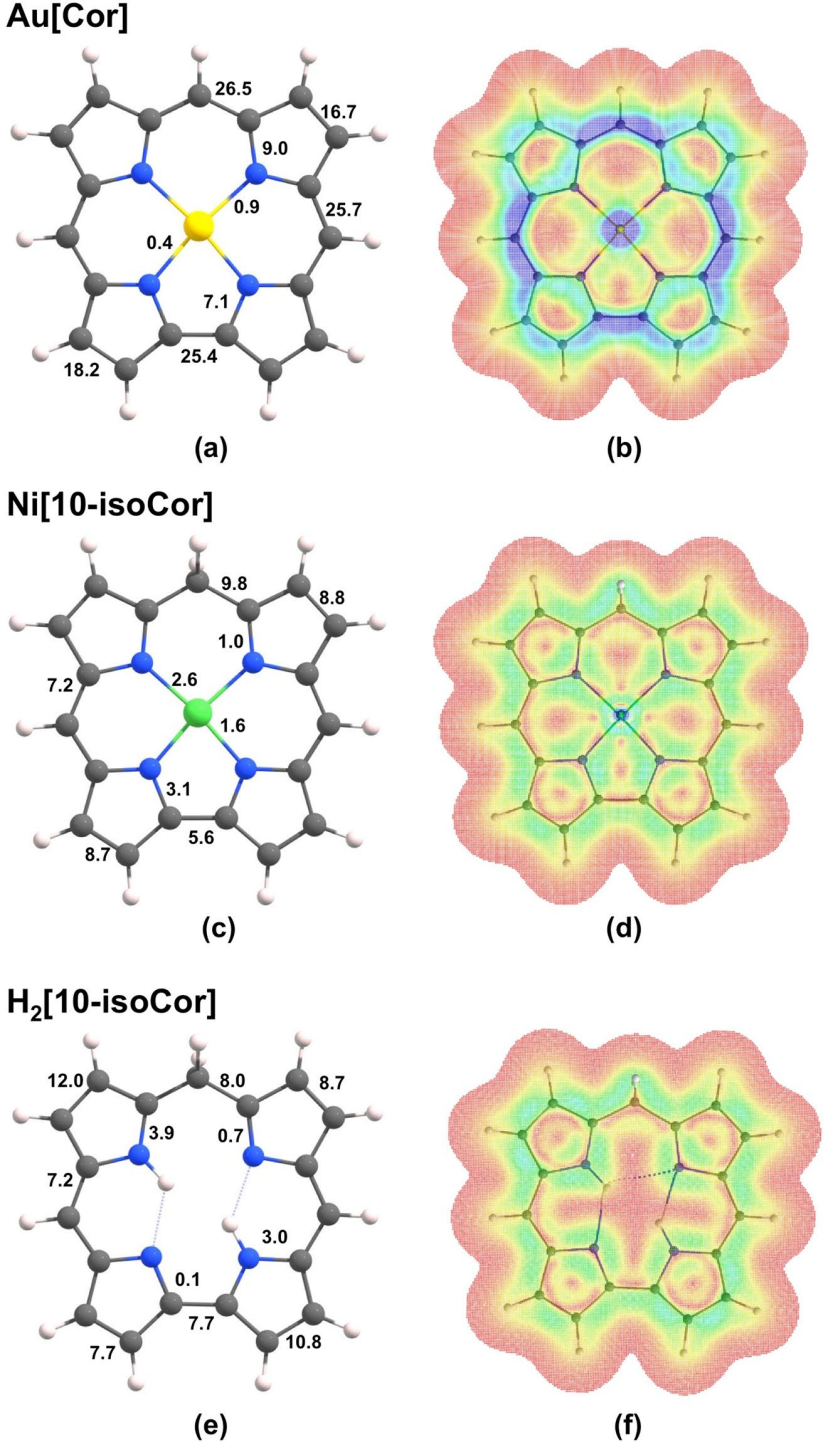
Current density pathways (**a**, **c**, and **e**) and plots (**b**, **d**, and **f**) for Au[Cor], Ni[10-isoCor], and H_2_[10-isoCor]. The plots refer to a displacement of 1 bohr above the molecular plane, where the π ring current is most intense. Colors ranging from blue (corresponding to 0.001 au) to red (0.0 au) represent stronger to weaker current densities.

Figure 5 depicts B3LYP/def2-TZVP current densities for unsubstituted gold corrole (Au[Cor])^10^ and free-base (H_2_[10-isoCor]) and nickel 10-isocorrole (Ni[10-isoCor]). Because the current density in all fully conjugated porphyrin-type molecules bifurcates at the pyrrole *α*-carbons, we will use the term ‘peripheral current’ to refer to the current along either the C9-C10 or the C1-C19 bond. The general features of the current density pathways for the molecules examined here are similar to those of other porphyrinoids; diatropic currents circulate along the outer rim of the molecules, while paratropic ones flow around the inner C_11_N_4_ framework^11,12^. Figure 5 shows that Au[Cor] sustains a strong diatropic peripheral current of ~26 nA·T^−1^ comparable to that of porphyrins. The current density passing between nitrogens and the central Au atom is almost negligible, reminiscent of current density pathways in porphyrins^11^. By comparison, the peripheral ring current in the unsubstituted metalloisocorrole Ni[10-isoCor] is ~9.8 nA·T^−1^ for the C9-C10 bond, which is about a third of that calculated for Au[Cor]. The reduced peripheral ring current in Ni[10-isoCor] is nevertheless far from insignificant and is just under that calculated for benzene (~11 nA·T^−1^). Qualitatively similar peripheral currents were also observed for the corresponding free-base isocorrole H_2_[10-isoCor] (Fig. 5). These data strongly suggest that Ni[10-isoCor] and H_2_[10-isoCor] are homoaromatic. Indeed, an examination of the π-type molecular orbitals of isocorrole derivatives provides conclusive proof of homoconjugation (hyperconjugative interactions); as discussed later in the paper, a total of 4 occupied MOs and the LUMO were found to exhibit with significant amplitudes at the saturated *meso* position.

**Figure 6:**
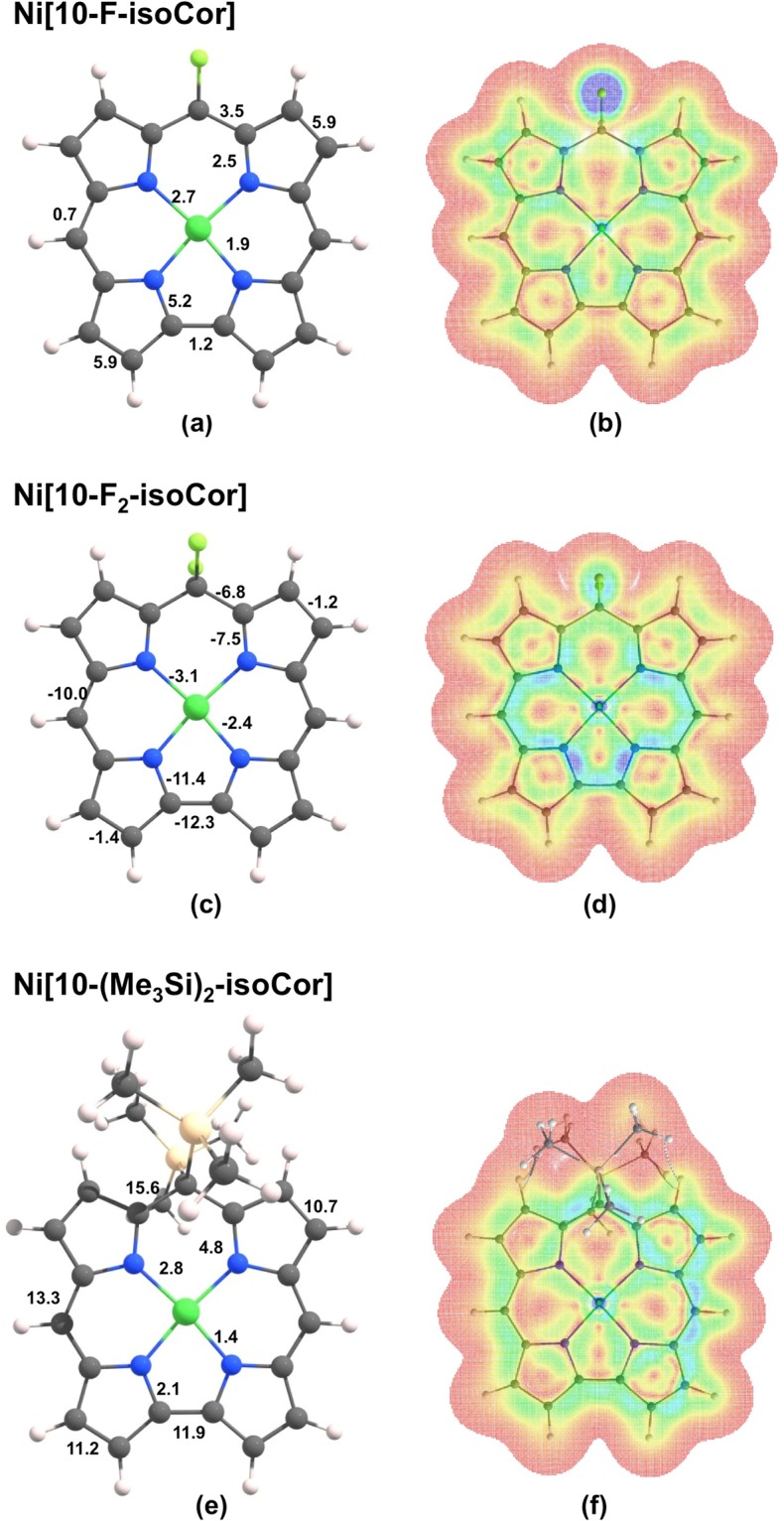
Integrated current densities (**a**, **c**, and **e**) and current density plots (**b**, **d**, and **f**) for Ni[10-F-isoCor], Ni[10-isoCor], and Ni[10-(Me_3_Si)_2_-isoCor]. The plots refer to a displacement of 1 bohr above the molecular plane. Colors ranging from blue (corresponding to 0.001 au) to red (0.0 au) represent stronger to weaker current densities. Negative values in entry (**c**) indicate net paratropic currents.

Remarkably, substituents at the saturated *meso* position C10 by fluoro and trimethylsilyl groups were found to result in striking changes in the calculated current densities (Fig. 6). Thus, fluoro substituents effectively quench the diatropic ring current; indeed, the difluorinated compound Ni[10-F_2_-isoCor] sustains a net paratropic peripheral current and is legitimately viewed as antihomoaromatic. The paratropic current in this compound flows largely around the 15-membered inner C_11_N_4_ ring, paralleling similar behavior observed for other antiaromatic porphyrinoids^13^. Trimethylsilyl groups on the other hand behave oppositely; the hypothetical bis(trimethylsilyl) compound Ni[10-(Me_3_Si)_2_-isoCor] sustains a greatly enhanced diatropic peripheral current and may be regarded as strongly homoaromatic. This diverse range of behavior is relatively simply attributed to the hyperconjugative effects of C-F σ* orbitals and of C-Si σ orbitals, as discussed by von Schleyer and coworkers^14,15^. Nevertheless, given that substituent effects on ring currents in aromatic systems are typically quite small^16–20^, the present dramatic variations as a function of substituents at the saturated *meso* carbon are unusual indeed.

### TDDFT calculations

**Figure 7:**
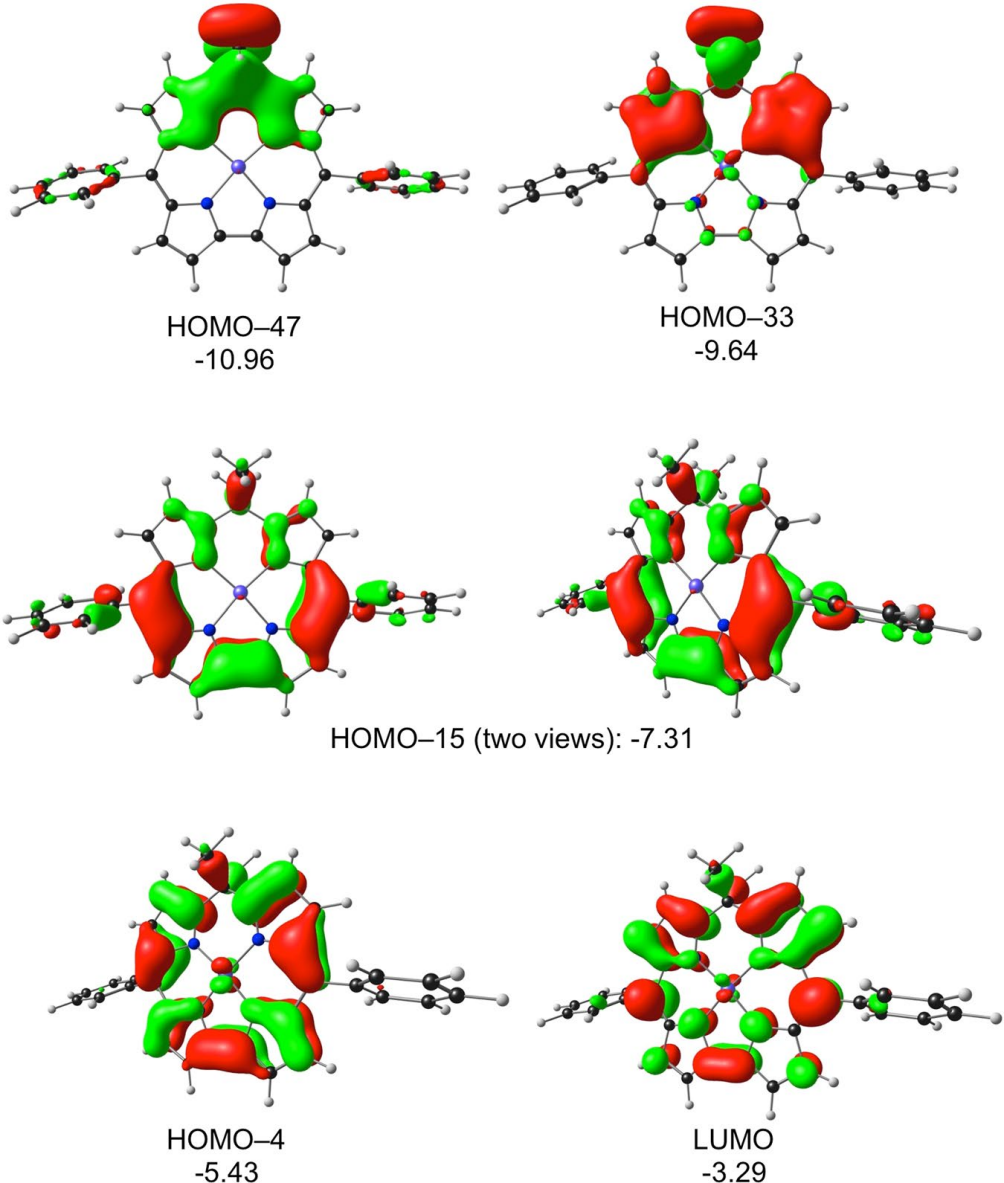
OLYP/STO-TZP π-type MOs of Ni[IsoPh_2_MeCor], which involve homoconjugative interactions at the C10 *meso* position, along with their orbital energies (eV).

**Figure 8:**
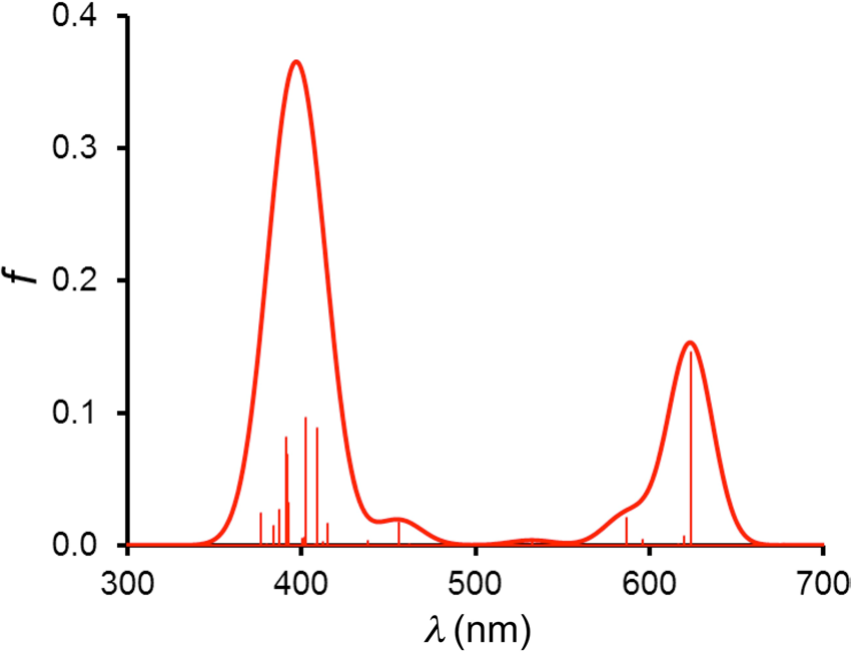
TDDFT oscillator strengths (*f*) plotted against wavelength (*λ*, nm) and an artificially broadened spectrum with Gaussians with FWHM = 30 nm.

**Figure 9:**
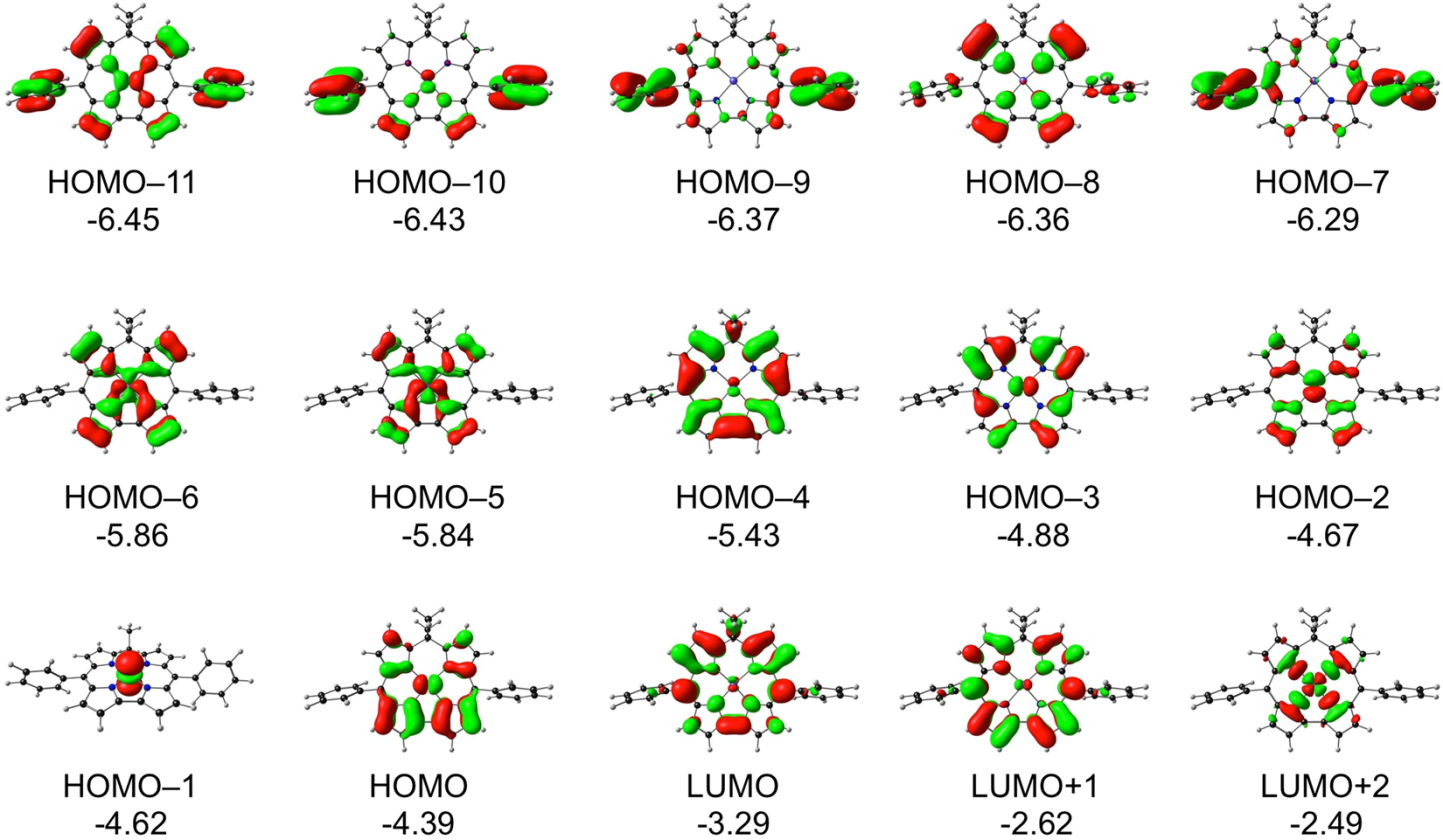
Selected OLYP/STO-TZP MOs relevant to Table 1, along with their orbital energies (eV).

Molecular orbital and TDDFT^21,22^ analyses were carried out on a number of isocorrole derivatives with all-electron OLYP/STO-TZP calculations. The various systems chosen yielded very similar qualitative insights; the discussion below is based on our results for nickel 10,10-dimethyl-5,15-diphenylisocorrole, Ni[iso-Ph_2_MeCor]. The ground-state calculations readily identified four π-type occupied MOs and the LUMO as having significant hyperconjugative interactions, i.e., relatively large amplitudes at the saturated *meso* position (Fig. 7). The TDDFT results (Table 1 and Figs 8 and 9) led to several additional insights. First, the energy spacing of the Kohn-Sham MO eigenvalues clearly does not correspond to Gouterman’s four-orbital model^23^. That said, the HOMO-4, HOMO-3, LUMO, and LUMO + 1 do resemble the four frontier orbitals of a porphyrin or corrole in terms of qualitative shape^24,25^. Of these, the HOMO-4 and LUMO exhibit significant hyperconjugative interactions, i.e., relatively large amplitudes at the saturated *meso* position. The most intense calculated transitions all involve substantial HOMO-1/HOMO → LUMO/LUMO + 1 character as well as smaller amounts of HOMO-4 character. The lowest-energy transition exhibits a Q-like transition energy of ~2.0 eV and has predominantly HOMO-3 → LUMO character. Furthermore, multiple transitions with a similar intensity then cluster in the typical Soret region (~3.0 eV), whose cumulative effect is a deceptively porphyrin-like overall spectrum. Finally, since the LUMO has large amplitudes at the *meso* positions and the majority of the low-energy transitions have significant LUMO character, it stands to reason that the UV-vis-NIR spectra should exhibit a strong dependence on *meso* substituents, as is indeed observed^1–5^.

**Table 1:** TDDFT (OLYP/STO-TZP) results for the main “Q” and “Soret” transitions of Ni[Iso10Me_2_–5,15Ph_2_C].

*E* (eV)	Symmetry	*λ* (nm)	*f*	From	To	% contribution
1.988	B	624	1.46 × 10^–1^	HOMO-3	LUMO	84.0
				HOMO-2	LUMO + 1	6.4
				HOMO	LUMO	3.9
				HOMO	LUMO + 2	2.7
				HOMO-3	LUMO + 2	0.6
3.033	A	409	8.87 × 10^–2^	HOMO	LUMO + 4	54.9
				HOMO-4	LUMO	10.0
				HOMO-3	LUMO + 1	8.9
				HOMO-8	LUMO	6.3
				HOMO-9	LUMO	5.4
3.081	A	402	9.63 × 10^–2^	HOMO	LUMO + 4	40.1
				HOMO-9	LUMO	27.8
				HOMO-4	LUMO	8.4
				HOMO-3	LUMO + 1	6.2
3.166	B	392	6.84 × 10^–2^	HOMO-4	LUMO + 1	37.7
				HOMO-7	LUMO	24.2
				HOMO-11	LUMO	16.0
				HOMO-14	LUMO	9.9
3.169	A	391	8.16 × 10^–2^	HOMO-9	LUMO	35.1
				HOMO-10	LUMO	19.4
				HOMO	LUMO + 6	9.4
				HOMO-4	LUMO	7.1
				HOMO-8	LUMO	5.6

## Conclusion

A first detailed DFT investigation has clearly implicated homoconjugation as a critical determinant of the observed spectroscopic features of isocorroles. Thus, the calculations indicated unsubstituted free-base 10-isocorrole and its nickel complex as clearly homoaromatic. That said, substituents at the saturated *meso* carbon were found to dramatically affect the homoconjugation. Thus, while fluoro substituents were found to quench the diatropic peripheral current, leading in some cases to net antihomoaromatic character, trimethylsilyl substituents were found to greatly enhance homoaromatic character. The calculations further revealed homoconjugative/hyperconjugative interactions in four π-type occupied MOs as well as in LUMO. The strong Soret-like feature of isocorroles was found to arise from the clustering of several near-degenerate transitions with individual Q-like intensities. Finally, the large amplitude of the LUMO at the *meso* positions provides a simple rationale for the observed large variations in the UV-vis-NIR spectral profiles of isocorroles as a function of *meso* substituents.

## Methods

All structures were fully optimized at B3LYP^26–28^/def2-TZVP^29^ computational level by Gaussian 09 rev. D1^30^. (All optimized Cartesian coordinates are listed in the Supplementary information.) Eigenvalues of the Hessian matrix of energy were checked to ensure that all structures correspond to local minima. To obtain current density plots and intensities GIAO NMR computations were performed at the same level of theory by Gaussian 09 rev. D1 and the wave function of the NMR computations were further analyzed by AIMAll (version 16.05.18) suite of programs^31^. The current density were obtained within the context of quantum theory of atoms in molecules as developed by Keith and Bader^32–36^. TDDFT calculations were performed with ADF2017^37,38^ on OLYP^27,39^/STO-TZP optimized geometries.

Free-base H_2_[iso-5/10-MeO-TPC] was synthesized according to the method described by the Kadish and Paolesse groups^2^. Although both isomeric free bases were isolated in reasonable yields, only the 10-methoxy compound (surprisingly) proved readily amenable to nickel insertion.

### Synthesis of H_2_[iso-5/10-MeO-TPC]

To a solution of 5,10,15-triphenylcorrole (46.7 mg) in a mixture of dichloromethane (20 mL) and methanol (10 mL) was added DDQ (20.4 mg, 1 eq) and the resulting solution was stirred for 10 min. The solvents were removed under vacuum and the solids were washed down through a plug of silica with dichloromethane. The two isomers were then separated with preparative thin-layer chromatography on silica plates employing 2:1 dichloromethane/hexane as solvent. Yields: 32 mg of the 5-isomer (64.8 %) and 5.5 mg (11.1%) of the 10-isomer.

### Spectroscopic data for H_2_[iso-5-MeO-TPC]

^1^H NMR (400 MHz, CDCl_3_, *δ*): 16.19 (s, 1H, NH), 15.85 (s, 1H, NH), 7.72 – 7.67 (m, 2H, 5-*o*-Ph), 7.53 – 7.48 (m, 2H, 15-*o*-Ph), 7.48 – 7.37 (m, 9H, 10-*o*-Ph and Ph), 7.25 – 7.22 (m, 2H, Ph), 6.93 (d, *J* = 4.6 Hz, 1H, *β*-H), 6.84 (d, *J* = 4.5 Hz, 1H, *β*-H), 6.56 (dd, *J* = 3.6, 2.6 Hz, 1H, *β*-H), 6.53 (d, *J* = 4.6 Hz, 1H, *β*-H), 6.50 (d, *J* = 4.6 Hz, 1H, *β*-H), 6.27 (dd, *J* = 4.3, 2.0 Hz, 1H, *β*-H), 6.11 (dd, *J* = 4.3, 2.6 Hz, 1H, *β*-H), 6.03 (dd, *J* = 3.6, 2.5 Hz, 1H, *β*-H), 3.43 (s, 3H, 5-MeO). UV-Vis (CH_2_Cl_2_) λ_max_ [nm; ϵ × 10^-4^ (M^-1^cm^-1^)]: 337 (2.42), 401 (3.93), 678 (0.60), 739 (0.56). MS (MALDI-TOF): m/z calcd for C_38_H_28_N_4_O 556.2263 [M^+^]; found 556.2272.

### Spectroscopic data for H_2_[iso-10-MeO-TPC]

^1^H NMR (400 MHz, CDCl_3_, *δ*): 15.58 (s, 2H, NH), 7.69 (d, *J* = 7.0 Hz, 2H, 10-*o*-Ph), 7.59 – 7.55 (m, 4H, 5,15-*o*-Ph), 7.48 – 7.42 (m, 6H, 5,15-*m*-Ph and 5,15-*p*-Ph), 7.25 – 7.16 (m, 3H, 10-*m*-Ph and 10-*p*-Ph), 6.69 – 6.67 (m, 4H, *β*-H), 6.61 (d, *J* = 4.3 Hz, 2H, *β*-H), 6.40 (d, *J* = 4.3 Hz, 2H, *β*-H), 3.49 (s, 3H, 10-MeO). UV-Vis (CH_2_Cl_2_) λ_max_ [nm; ϵ × 10^-4^ (M^-1^cm^-1^)]: 351 (2.24), 430 (4.09), 668 (0.49), 721 (0.53). MS (MALDI-TOF): m/z calcd for C_38_H_28_N_4_O 556.2263 [M^+^]; found: 556.2272.

### Synthesis of Ni[iso-5/10-MeO-TPC]

Free-base isocorrole (12.8 mg, mixture of isomers) and Ni(OAc)_2_ ∙ 4H_2_O (48.9 mg, 6 eq) were dissolved in dry DMF (5 ml) and refluxed for 1 h. The solvent was removed under vacuum and the solids were washed down with dichloromethane through a silica gel plug. The resulting product, upon preparative thin-layer chromatography on a silica plate with 2:1 dichloromethane/hexane as eluent, yielded a brown band composed of Ni[5,10,15-triphenyl-10-methoxyisocorrole]. Yield 1.2 mg (8.5%).

### Spectroscopic data for Ni[iso-10-MeO-TPC]

^1^H NMR (400 MHz, CDCl_3_, *δ*): 7.86 (d, *J* = 7.6 Hz, 2H, Ph), 7.45 – 7.35 (m, 13H, Ph), 6.41 (d, *J* = 4.5 Hz, 2H, *β*-H), 6.27 – 6.23 (m, 4H, *β*-H), 6.15 (d, *J* = 4.5 Hz, 2H, *β*-H), 3.39 (s, 3H, 10-MeO). UV-Vis (CH_2_Cl_2_) λ_max_ [nm; ϵ × 10^-4^ (M^-1^cm^-1^)]: 356 (1.23), 430 (2.51), 533 (0.45), 818 (0.18), 909 (0.39); MS (MALDI-TOF): m/z calcd for C_38_H_26_N_4_ONi: 612.1460 [M^+^]; found 612.1638.

## Electronic supplementary material

Supplemenatry information
