# Supplementary material for: Isocorroles as Homoaromatic NIR-Absorbing Chromophores: A First Quantum Chemical Study
Source: Sci Rep. 2018 Aug 10;8:11952. doi: 10.1038/s41598-018-29819-3 (PMC6086901; doi:10.1038/s41598-018-29819-3)
Supplement: Supplementary file 1 — Supplemenatry information [file 41598_2018_29819_MOESM1_ESM.pdf]

Supplementary Information

Isocorroles as an Emerging Class of Homoaromatic NIR-Absorbing Chromophores. A First Quantum Chemical Study

Cina Foroutan-Nejad,*,[a] Simon Larsen,[b] Jeanet Conradie,*,[b,c] Abhik Ghosh,*,[b]

[a] CEITEC – Central European Institute of Technology, Masaryk University, Kamenice 5, CZ – 62500 Brno, Czech Republic; E-mail: cina.foroutannejad@ceitc.muni.cz (CFN)

[b] Department of Chemistry, UiT – The Arctic University of Norway, 9037 Tromsø, Norway; E-mail: abhik.ghosh@uit.no (AG)

[c] Department of Chemistry, University of the Free State, 9300 Bloemfontein, Republic of South Africa; E-mail: conradj@ufs.ac.nz

Optimized Cartesian coordinates (Å)

Table of Contents

1. Au[Cor] B3LYP/def2-TZVP S2
2. H_2[Cor] B3LYP/def2-TZVP S2
3. H_2[10-isoCor] B3LYP/def2-TZVP S3
4. H_2[10-Si(Me)$_2$-isoCor] B3LYP/def2-TZVP S4
5. H_2[10-phenyl$_2$-isoCor] B3LYP/def2-TZVP S5
6. H_2[10-methoxy,10-phenyl-isoCor] B3LYP/def2-TZVP S6
7. H_2[10-Si(Me)$_2$-isoCor] B3LYP/def2-TZVP S7
8. Ni[10-methoxy,10-H-isoCor] B3LYP/def2-TZVP S8
9. Ni[10-methoxy,10-phenyl-isoCor] B3LYP/def2-TZVP S9
10. Ni[10-phenyl$_2$-isoCor] B3LYP/def2-TZVP S10
11. Ni[10-F-isoCor] B3LYP/def2-TZVP S11
12. Ni[10-F$_2$-isoCor] B3LYP/def2-TZVP S12
13. Ni[10-(Me$_3$Si)$_2$-isoCor] B3LYP/def2-TZVP S13
14. Ni[10-Si(Me)$_2$-isoCor] B3LYP/def2-TZVP S14
15. Ni[iso-10Me$_2$-5,15Ph$_2$C] OLYP/STO-TZP S15
16. Ni[iso-10Me,10H-5,15Ph$_2$C] B3LYP/def2-TZVP S16
17. Ni[iso-10Me$_2$-5,15Ph$_2$C] B3LYP/def2-TZVP S17
18. Ni[10-isoCor] B3LYP/def2-TZVP S18

1. Au[Cor] B3LYP/def2-TZVP

C	3.629586000	-1.828738000	0.000130000
C	2.744816000	-0.715797000	0.000044000
C	2.860635000	-2.984364000	0.000205000
C	2.744816000	0.715795000	-0.000075000
N	1.484633000	-1.250769000	0.000093000
C	1.478768000	-2.609694000	0.000195000
C	3.629587000	1.828736000	-0.000187000
C	0.268524000	-3.330814000	0.000256000
N	1.484634000	1.250768000	-0.000105000
C	1.478770000	2.609694000	-0.000221000
C	0.268526000	3.330813000	-0.000281000
C	2.860637000	2.984362000	-0.000230000
C	-1.024657000	2.792060000	-0.000240000
N	-1.346246000	1.448922000	-0.000126000
C	-2.282466000	3.489185000	-0.000295000
C	-3.292626000	2.566932000	-0.000231000
C	-2.699945000	1.256290000	-0.000110000
C	-1.024659000	-2.792059000	0.000217000
N	-1.346247000	-1.448922000	0.000121000
C	-2.282468000	-3.489184000	0.000268000
C	-3.292627000	-2.566930000	0.000187000
C	-2.699946000	-1.256289000	0.000098000
H	4.706438000	-1.778997000	0.000122000
H	3.230766000	-3.997304000	0.000270000
H	4.706439000	1.778994000	-0.000205000
H	0.335144000	-4.410812000	0.000333000
H	0.335147000	4.410811000	-0.000365000
H	3.230768000	3.997302000	-0.000296000
H	-2.384239000	4.563213000	-0.000382000
H	-4.353328000	2.761975000	-0.000254000
H	-2.384242000	-4.563212000	0.000349000
H	-4.353329000	-2.761972000	0.000192000
Au	-0.010848000	0.000000000	0.000025000
C	-3.322829000	0.000001000	-0.000001000
H	-4.404669000	0.000001000	-0.000012000

2. H_2[Cor] B3LYP/def2-TZVP

C	-3.753863000	1.799453000	0.000142000
C	-2.823069000	0.723823000	0.000056000
C	-3.005833000	2.961247000	0.000282000
C	-2.823071000	-0.723810000	-0.000066000
N	-1.575377000	1.214462000	0.000100000
C	-1.627828000	2.578367000	0.000219000
C	-3.753873000	-1.799436000	-0.000126000
C	-0.451315000	3.314123000	0.000272000

N	-1.575384000	-1.214455000	-0.000113000
C	-1.627841000	-2.578362000	-0.000220000
C	-0.451333000	-3.314120000	-0.000260000
C	-3.005851000	-2.961233000	-0.000299000
C	0.834075000	-2.764028000	-0.000210000
N	1.120079000	-1.406572000	-0.000124000
C	2.062962000	-3.485578000	-0.000245000
C	3.079531000	-2.562157000	-0.000146000
C	2.457062000	-1.288196000	-0.000088000
C	0.834088000	2.764029000	0.000220000
N	1.120084000	1.406568000	0.000118000
C	2.062984000	3.485568000	0.000245000
C	3.079543000	2.562141000	0.000178000
C	2.457062000	1.288182000	0.000084000
H	-4.828244000	1.715651000	0.000125000
H	-3.377048000	3.973878000	0.000391000
H	-4.828253000	-1.715628000	-0.000092000
H	-0.520498000	4.395218000	0.000365000
H	-0.520516000	-4.395215000	-0.000351000
H	-3.377072000	-3.973862000	-0.000415000
H	2.149006000	-4.560972000	-0.000323000
H	4.143024000	-2.728327000	-0.000131000
H	2.149034000	4.560962000	0.000320000
H	4.143038000	2.728299000	0.000182000
H	0.427970000	0.643758000	0.000051000
C	3.184406000	-0.000009000	-0.000010000
H	4.264406000	-0.000005000	0.000004000
H	-0.792791000	-0.544792000	-0.000054000

3. H_2[10-isoCor] B3LYP/def2-TZVP

C	0.144289570	3.996224096	-0.000235850
C	-0.409026282	2.661114437	-0.001871308
C	1.504489771	3.840884527	0.006650465
C	-1.746335961	2.110538541	-0.016283081
N	0.559004595	1.755386998	0.007565775
C	1.757065490	2.415357220	0.010955403
C	-3.091844821	2.540775593	0.024022372
C	2.941669863	1.696210551	0.011023418
N	-1.782184407	0.766314693	-0.053594519
C	-3.042896412	0.270171654	-0.025548573
C	-3.209313306	-1.133998179	-0.016853316
C	-3.898030770	1.398462585	0.018050684
C	-2.174858548	-2.053845035	-0.011434268
N	-0.808763619	-1.723338463	-0.012849525
C	-2.302315440	-3.489531794	0.007286771
C	-1.037727238	-3.996397782	0.018481403
C	-0.143679613	-2.856481539	0.005551158
C	3.030829223	0.287707701	0.011107350
N	1.927565468	-0.547262236	0.032624609

C	4.154618954	-0.561503428	-0.026022926
C	3.696196963	-1.876568773	-0.031264088
C	2.289903518	-1.839203837	0.005966850
H	-0.407891656	4.922870382	-0.007472795
H	2.249480833	4.621972208	0.007831561
H	-3.428939745	3.563418353	0.060223350
H	3.880342036	2.237267056	0.003453220
H	-4.220045778	-1.523132839	-0.003786396
H	-4.975411666	1.373386034	0.048738148
H	-3.232649266	-4.036653984	0.011925112
H	-0.743160135	-5.034796006	0.035236973
H	5.181134005	-0.232783066	-0.051495301
H	4.295823479	-2.772020669	-0.059046209
C	1.352913367	-3.008897096	0.022819005
H	1.621715481	-3.655317698	-0.822356177
H	1.600964594	-3.615324286	0.904156497
H	-0.975186065	0.152411870	-0.077811345
H	0.988779654	-0.165120977	0.059770167

4. H_2[10-Si(Me)$_2$-isoCor] B3LYP/def2-TZVP

C	-3.788984000	-1.722046000	-0.004446000
C	-2.808364000	-0.708648000	-0.002122000
C	-3.121005000	-2.948800000	-0.006572000
C	-2.766338000	0.735085000	0.000474000
N	-1.611065000	-1.325584000	-0.002808000
C	-1.733617000	-2.679652000	-0.005534000
C	-3.794791000	1.752872000	0.002054000
C	-0.571670000	-3.481906000	-0.006868000
N	-1.560197000	1.277520000	0.001704000
C	-1.721890000	2.642439000	0.004106000
C	-0.625481000	3.479865000	0.005885000
C	-3.136786000	2.949869000	0.004341000
C	0.724512000	3.055807000	0.005467000
N	1.112318000	1.732406000	0.003108000
C	1.910960000	3.810434000	0.007293000
C	2.979276000	2.914552000	0.006021000
C	2.459893000	1.604517000	0.003401000
C	0.725902000	-3.006902000	-0.005685000
N	1.074404000	-1.647302000	-0.002880000
C	1.928853000	-3.799435000	-0.007168000
C	2.971089000	-2.923098000	-0.005293000
C	2.397142000	-1.585469000	-0.002621000
H	-4.855327000	-1.568825000	-0.004594000
H	-3.571451000	-3.928241000	-0.008676000
H	-4.861039000	1.589806000	0.001531000
H	-0.710028000	-4.556476000	-0.009082000
H	-0.792017000	4.550515000	0.007806000
H	-3.573644000	3.936808000	0.006008000
H	1.963386000	4.887511000	0.009338000

H	4.026519000	3.171044000	0.006901000
H	1.970528000	-4.878065000	-0.009367000
H	4.023318000	-3.164730000	-0.005700000
H	0.413615000	0.996705000	0.001467000
Si	3.442320000	-0.009736000	0.000935000
C	4.525145000	-0.022754000	1.540296000
H	5.157518000	-0.913095000	1.565547000
H	5.177595000	0.852924000	1.566094000
H	3.917668000	-0.016096000	2.447060000
C	4.527015000	-0.016986000	-1.537145000
H	5.179459000	0.858807000	-1.558898000
H	5.159469000	-0.907196000	-1.564927000
H	3.920648000	-0.006996000	-2.444620000
H	-0.696793000	-0.880383000	-0.001587000

5. H_2[10-phenyl$_2$-isoCor] B3LYP/def2-TZVP

C	-3.110132000	-0.475333000	5.359322000
C	-1.735029000	-0.464269000	4.914403000
C	-3.871129000	-0.307416000	4.233746000
C	-0.448288000	-0.493829000	5.578741000
N	-1.668390000	-0.313701000	3.601251000
C	-2.946575000	-0.193068000	3.125953000
C	0.086632000	-0.462117000	6.883854000
C	-3.154384000	0.075139000	1.786040000
N	0.597937000	-0.402967000	4.737287000
C	1.778930000	-0.255813000	5.384618000
C	2.930779000	0.025431000	4.613877000
C	1.473568000	-0.314296000	6.764327000
C	2.957400000	0.187699000	3.241610000
N	1.856136000	0.048543000	2.375877000
C	4.111644000	0.559269000	2.464926000
C	3.704880000	0.643442000	1.170736000
C	2.290755000	0.311968000	1.162808000
C	-2.136212000	0.233670000	0.818889000
N	-0.782431000	0.112606000	1.082296000
C	-2.235389000	0.544319000	-0.548997000
C	-0.945530000	0.604937000	-1.069268000
C	-0.046353000	0.326754000	-0.024968000
H	-3.458865000	-0.585331000	6.374155000
H	-4.947682000	-0.255591000	4.176818000
H	-0.476123000	-0.518153000	7.800825000
H	-4.170541000	0.195257000	1.429137000
H	3.866031000	0.165261000	5.142297000
H	2.183089000	-0.235598000	7.572151000
H	5.098827000	0.746908000	2.858785000
H	4.299930000	0.925677000	0.318872000
H	-3.157215000	0.714384000	-1.081908000
H	-0.671725000	0.827421000	-2.085492000
C	1.467638000	0.148421000	-0.131818000

C	1.924161000	1.206319000	-1.164954000
C	2.121247000	0.911713000	-2.512175000
C	2.048806000	2.536872000	-0.754988000
C	2.452053000	1.913997000	-3.421265000
H	2.015398000	-0.104371000	-2.864980000
C	2.380591000	3.536996000	-1.657499000
H	1.881592000	2.793643000	0.283620000
C	2.587375000	3.228635000	-2.998562000
H	2.602325000	1.659300000	-4.463076000
H	2.474692000	4.559353000	-1.313303000
H	2.848253000	4.006810000	-3.704662000
C	1.774103000	-1.315675000	-0.585536000
C	3.057973000	-1.669915000	-1.013255000
C	0.808835000	-2.319693000	-0.521244000
C	3.361167000	-2.976036000	-1.371993000
H	3.833541000	-0.920585000	-1.074846000
C	1.112228000	-3.630853000	-0.876091000
H	-0.194711000	-2.091903000	-0.192959000
C	2.388277000	-3.966891000	-1.304212000
H	4.363842000	-3.218539000	-1.701566000
H	0.340566000	-4.388118000	-0.815809000
H	2.623904000	-4.986631000	-1.581062000
H	0.534132000	-0.343669000	3.727824000
H	-0.443231000	-0.086210000	2.015366000

6. H_2[10-methoxy,10-phenyl-isoCor] B3LYP/def2-TZVP

C	-3.518199000	-1.711220000	-0.442144000
C	-2.533907000	-0.689094000	-0.156627000
C	-2.829922000	-2.891396000	-0.496986000
C	-2.556911000	0.761454000	-0.195280000
N	-1.333975000	-1.221680000	-0.005423000
C	-1.439498000	-2.569607000	-0.249089000
C	-3.463944000	1.795663000	-0.503555000
C	-0.297721000	-3.329216000	-0.408422000
N	-1.343742000	1.335202000	-0.089416000
C	-1.369502000	2.661622000	-0.376460000
C	-0.137529000	3.343267000	-0.534004000
C	-2.725215000	2.979819000	-0.616721000
C	1.111339000	2.767106000	-0.408324000
N	1.339461000	1.441188000	-0.004631000
C	2.381147000	3.384904000	-0.701095000
C	3.339985000	2.440476000	-0.483033000
C	2.636710000	1.255587000	-0.040837000
C	1.027616000	-2.835534000	-0.314551000
N	1.338256000	-1.554710000	0.099470000
C	2.245489000	-3.427129000	-0.685501000
C	3.256645000	-2.477227000	-0.504468000
C	2.659592000	-1.313192000	-0.006042000
H	-4.573329000	-1.560384000	-0.607887000

H	-3.229456000	-3.870526000	-0.712931000
H	-4.525482000	1.684822000	-0.650166000
H	-0.401540000	-4.368692000	-0.696263000
H	-0.176710000	4.384099000	-0.831903000
H	-3.110014000	3.955649000	-0.865597000
H	2.521057000	4.397000000	-1.049354000
H	4.404374000	2.526125000	-0.617412000
H	2.360778000	-4.428264000	-1.068799000
H	4.302052000	-2.604373000	-0.720783000
C	3.309239000	-0.022308000	0.485170000
C	3.294385000	0.042518000	2.036111000
C	3.644288000	1.234154000	2.678158000
C	2.997283000	-1.069173000	2.822862000
C	3.691905000	1.310828000	4.062941000
H	3.877997000	2.112122000	2.091299000
C	3.039959000	-0.992040000	4.211981000
H	2.729196000	-2.008026000	2.359251000
C	3.386581000	0.196833000	4.837893000
H	3.962439000	2.245863000	4.537457000
H	2.799819000	-1.867971000	4.801560000
H	3.416768000	0.257766000	5.918454000
H	-0.465215000	0.844031000	0.042376000
H	0.604030000	-0.898402000	0.338347000
O	4.648157000	0.034182000	-0.025542000
C	5.691800000	-0.597013000	0.704902000
H	6.556352000	-0.587333000	0.042257000
H	5.935757000	-0.051177000	1.619320000
H	5.457989000	-1.630680000	0.969323000

7. H_2[10-Si(Me)$_2$-isoCor] B3LYP/def2-TZVP

C	-3.788984000	-1.722046000	-0.004446000
C	-2.808364000	-0.708648000	-0.002122000
C	-3.121005000	-2.948800000	-0.006572000
C	-2.766338000	0.735085000	0.000474000
N	-1.611065000	-1.325584000	-0.002808000
C	-1.733617000	-2.679652000	-0.005534000
C	-3.794791000	1.752872000	0.002054000
C	-0.571670000	-3.481906000	-0.006868000
N	-1.560197000	1.277520000	0.001704000
C	-1.721890000	2.642439000	0.004106000
C	-0.625481000	3.479865000	0.005885000
C	-3.136786000	2.949869000	0.004341000
C	0.724512000	3.055807000	0.005467000
N	1.112318000	1.732406000	0.003108000
C	1.910960000	3.810434000	0.007293000
C	2.979276000	2.914552000	0.006021000
C	2.459893000	1.604517000	0.003401000
C	0.725902000	-3.006902000	-0.005685000
N	1.074404000	-1.647302000	-0.002880000

C	1.928853000	-3.799435000	-0.007168000
C	2.971089000	-2.923098000	-0.005293000
C	2.397142000	-1.585469000	-0.002621000
H	-4.855327000	-1.568825000	-0.004594000
H	-3.571451000	-3.928241000	-0.008676000
H	-4.861039000	1.589806000	0.001531000
H	-0.710028000	-4.556476000	-0.009082000
H	-0.792017000	4.550515000	0.007806000
H	-3.573644000	3.936808000	0.006008000
H	1.963386000	4.887511000	0.009338000
H	4.026519000	3.171044000	0.006901000
H	1.970528000	-4.878065000	-0.009367000
H	4.023318000	-3.164730000	-0.005700000
H	0.413615000	0.996705000	0.001467000
Si	3.442320000	-0.009736000	0.000935000
C	4.525145000	-0.022754000	1.540296000
H	5.157518000	-0.913095000	1.565547000
H	5.177595000	0.852924000	1.566094000
H	3.917668000	-0.016096000	2.447060000
C	4.527015000	-0.016986000	-1.537145000
H	5.179459000	0.858807000	-1.558898000
H	5.159469000	-0.907196000	-1.564927000
H	3.920648000	-0.006996000	-2.444620000
H	-0.696793000	-0.880383000	-0.001587000

8. Ni[10-methoxy,10-H-isoCor] B3LYP/def2-TZVP

C	-3.204683000	-0.008994000	5.342801000
C	-1.828717000	0.001131000	4.987615000
C	-3.914830000	-0.020391000	4.156320000
C	-0.540054000	0.001118000	5.624963000
N	-1.717902000	-0.001258000	3.646079000
C	-2.961959000	-0.016651000	3.092333000
C	0.012772000	-0.009063000	6.934094000
C	-3.104187000	-0.034245000	1.707316000
N	0.458884000	-0.001268000	4.722678000
C	1.654031000	-0.016678000	5.375315000
C	2.841104000	-0.034292000	4.647747000
C	1.386745000	-0.020327000	6.778380000
C	2.918855000	-0.032393000	3.260627000
N	1.827009000	-0.004917000	2.386774000
C	4.111187000	-0.049211000	2.482407000
C	3.737807000	-0.027914000	1.163291000
C	2.313987000	0.000000000	1.144454000
C	-2.048954000	-0.032366000	0.803642000
N	-0.691781000	-0.004905000	1.141027000
C	-2.154117000	-0.049296000	-0.616294000
C	-0.879128000	-0.027863000	-1.120159000
C	0.000000000	0.000000000	0.000000000
H	-3.606595000	-0.009292000	6.342909000

H	-4.987174000	-0.030925000	4.039947000
H	-0.538138000	-0.009401000	7.860511000
H	-4.104516000	-0.051870000	1.292153000
H	3.778186000	-0.051881000	5.190790000
H	2.130069000	-0.030800000	7.560003000
H	5.112000000	-0.070053000	2.884717000
H	4.375783000	-0.017136000	0.294875000
H	-3.081276000	-0.070203000	-1.167524000
H	-0.576156000	-0.017048000	-2.154260000
Ni	0.000000000	0.000000000	2.911567000
C	1.496984000	0.013191000	-0.115204000
H	1.755597000	-0.917690000	-0.638103000
C	1.953026000	1.150743000	-1.037280000
C	2.557903000	0.876226000	-2.260253000
C	1.766524000	2.481701000	-0.660224000
C	2.971413000	1.910497000	-3.096337000
H	2.708800000	-0.152992000	-2.565328000
C	2.177586000	3.513391000	-1.491357000
H	1.296894000	2.708747000	0.289297000
C	2.782256000	3.231186000	-2.713915000
H	3.440478000	1.679891000	-4.044719000
H	2.026458000	4.541155000	-1.185821000
H	3.102553000	4.037434000	-3.361527000

9. Ni[10-methoxy,10-phenyl-isoCor] B3LYP/def2-TZVP

C	-3.633619000	-1.690857000	-0.304992000
C	-2.682099000	-0.643532000	-0.161863000
C	-2.913395000	-2.870278000	-0.348363000
C	-2.640227000	0.799331000	-0.175821000
N	-1.449080000	-1.172410000	-0.097157000
C	-1.531168000	-2.527572000	-0.229955000
C	-3.525839000	1.897101000	-0.339840000
C	-0.367524000	-3.284411000	-0.312041000
N	-1.375823000	1.254746000	-0.122854000
C	-1.377067000	2.607894000	-0.283083000
C	-0.167076000	3.293193000	-0.386226000
C	-2.734381000	3.031008000	-0.407611000
C	1.087984000	2.705592000	-0.285795000
N	1.323877000	1.357957000	-0.004132000
C	2.347526000	3.356414000	-0.417878000
C	3.321879000	2.412187000	-0.202576000
C	2.643583000	1.192910000	0.070607000
C	0.924779000	-2.771256000	-0.218692000
N	1.245501000	-1.435194000	0.028705000
C	2.136581000	-3.505771000	-0.313912000
C	3.170235000	-2.620410000	-0.108598000
C	2.576176000	-1.350977000	0.117032000
H	-4.703012000	-1.577444000	-0.376906000
H	-3.306498000	-3.868343000	-0.462015000

H	-4.600279000	1.847801000	-0.409154000
H	-0.458566000	-4.352601000	-0.468101000
H	-0.195183000	4.360397000	-0.570217000
H	-3.066705000	4.048465000	-0.541274000
H	2.483572000	4.400484000	-0.653544000
H	4.389758000	2.536937000	-0.239336000
H	2.208210000	-4.563083000	-0.516358000
H	4.222805000	-2.838740000	-0.123292000
C	3.292899000	-0.088570000	0.566486000
C	3.306670000	-0.005523000	2.122673000
C	3.934746000	1.078890000	2.743063000
C	2.723606000	-0.980757000	2.929827000
C	3.982982000	1.180947000	4.125872000
H	4.385373000	1.854756000	2.140366000
C	2.765575000	-0.875314000	4.317359000
H	2.227638000	-1.831518000	2.486349000
C	3.394978000	0.203295000	4.921595000
H	4.475019000	2.030871000	4.582145000
H	2.301858000	-1.644130000	4.922613000
H	3.425972000	0.285141000	6.000716000
O	4.618330000	-0.069168000	0.021295000
C	5.684180000	-0.682925000	0.736799000
H	6.454201000	-0.894861000	-0.004529000
H	6.095732000	-0.014978000	1.497523000
H	5.391934000	-1.614360000	1.223686000
Ni	0.000000000	0.000000000	0.000000000

10. Ni[10-phenyl$_2$-isoCor] B3LYP/def2-TZVP

C	-3.204459000	0.033551000	5.357196000
C	-1.829710000	0.011549000	5.001019000
C	-3.915254000	0.054568000	4.170474000
C	-0.540015000	-0.004636000	5.638101000
N	-1.718114000	0.017286000	3.660105000
C	-2.962602000	0.045233000	3.107748000
C	0.013087000	-0.004994000	6.945923000
C	-3.100493000	0.061973000	1.723198000
N	0.456976000	-0.009194000	4.734080000
C	1.652085000	-0.007737000	5.386394000
C	2.835693000	0.005704000	4.653756000
C	1.387884000	-0.006719000	6.788681000
C	2.911103000	0.010085000	3.267713000
N	1.823491000	-0.008787000	2.385263000
C	4.107284000	0.038080000	2.498000000
C	3.742952000	0.032154000	1.179169000
C	2.317560000	0.000000000	1.144945000
C	-2.045660000	0.048574000	0.819967000
N	-0.684177000	0.017319000	1.147133000
C	-2.162090000	0.049664000	-0.597432000
C	-0.893382000	0.020007000	-1.110498000

C	0.000000000	0.000000000	0.000000000
H	-3.605621000	0.034266000	6.357576000
H	-4.987438000	0.075509000	4.054150000
H	-0.537085000	-0.002780000	7.872744000
H	-4.099376000	0.083413000	1.304603000
H	3.775428000	0.017351000	5.192458000
H	2.132341000	-0.006192000	7.569272000
H	5.105677000	0.059453000	2.906190000
H	4.393772000	0.051123000	0.322772000
H	-3.093029000	0.073608000	-1.142098000
H	-0.609041000	0.014862000	-2.147928000
Ni	0.000000000	0.000000000	2.920012000
C	1.512025000	-0.059262000	-0.143149000
C	1.877507000	1.171324000	-1.021238000
C	1.892815000	1.121059000	-2.415709000
C	2.096233000	2.406801000	-0.406747000
C	2.138547000	2.263392000	-3.170265000
H	1.713325000	0.186264000	-2.927397000
C	2.337772000	3.549194000	-1.158156000
H	2.075833000	2.481136000	0.672528000
C	2.364977000	3.482880000	-2.546232000
H	2.150011000	2.194670000	-4.251005000
H	2.505526000	4.492954000	-0.654540000
H	2.558098000	4.371445000	-3.133726000
C	1.898086000	-1.432093000	-0.775207000
C	3.045649000	-1.594555000	-1.554179000
C	1.141291000	-2.567714000	-0.479089000
C	3.412117000	-2.845258000	-2.037202000
H	3.664197000	-0.741989000	-1.794831000
C	1.509119000	-3.819567000	-0.956579000
H	0.254659000	-2.480272000	0.134012000
C	2.645001000	-3.965212000	-1.742793000
H	4.304248000	-2.940661000	-2.643701000
H	0.901669000	-4.681849000	-0.711783000
H	2.930183000	-4.939193000	-2.119616000

11. Ni[10-F-isoCor] B3LYP/def2-TZVP

C	-3.599782000	-1.796450000	-0.057947000
C	-2.670589000	-0.720657000	-0.086548000
C	-2.851528000	-2.957718000	-0.011394000
C	-2.670695000	0.720275000	-0.086434000
N	-1.420518000	-1.214784000	-0.068052000
C	-1.474363000	-2.575807000	-0.012719000
C	-3.600046000	1.795925000	-0.057658000
C	-0.298227000	-3.315128000	0.063453000
N	-1.420697000	1.214582000	-0.067859000
C	-1.474742000	2.575589000	-0.012308000
C	-0.298716000	3.315071000	0.063980000
C	-2.851964000	2.957296000	-0.010920000

C	0.982164000	2.769948000	0.065822000
N	1.273671000	1.407747000	-0.025597000
C	2.210618000	3.483173000	0.154438000
C	3.227751000	2.559183000	0.106983000
C	2.602005000	1.291323000	-0.005289000
C	0.982572000	-2.769817000	0.065384000
N	1.273879000	-1.407557000	-0.025821000
C	2.211132000	-3.482874000	0.153888000
C	3.228128000	-2.558726000	0.106587000
C	2.602195000	-1.290941000	-0.005491000
H	-4.674308000	-1.713489000	-0.066596000
H	-3.223044000	-3.969705000	0.024831000
H	-4.674561000	1.712807000	-0.066319000
H	-0.371864000	-4.393707000	0.131346000
H	-0.372512000	4.393628000	0.132044000
H	-3.223629000	3.969223000	0.025468000
H	2.299407000	4.554538000	0.244397000
H	4.289781000	2.732529000	0.150324000
H	2.300078000	-4.554240000	0.243677000
H	4.290184000	-2.731922000	0.149906000
Ni	-0.003787000	0.000003000	-0.063379000
C	3.326031000	0.000261000	-0.222732000
H	3.664448000	0.000368000	-1.270117000
F	4.501991000	0.000289000	0.539037000

12. Ni[10-F_2-isoCor] B3LYP/def2-TZVP

C	-3.603975000	-1.797756000	-0.000137000
C	-2.674270000	-0.722826000	-0.000054000
C	-2.855379000	-2.960233000	-0.000292000
C	-2.674374000	0.722430000	0.000068000
N	-1.425331000	-1.214474000	-0.000100000
C	-1.478965000	-2.577722000	-0.000222000
C	-3.604240000	1.797224000	0.000119000
C	-0.301913000	-3.315933000	-0.000276000
N	-1.425510000	1.214262000	0.000116000
C	-1.479345000	2.577505000	0.000221000
C	-0.302403000	3.315885000	0.000254000
C	-2.855817000	2.959810000	0.000315000
C	0.980641000	2.771300000	0.000204000
N	1.272991000	1.407039000	0.000124000
C	2.206353000	3.492759000	0.000240000
C	3.226259000	2.570411000	0.000129000
C	2.600986000	1.300707000	0.000083000
C	0.981049000	-2.771163000	-0.000224000
N	1.273196000	-1.406856000	-0.000118000
C	2.206871000	-3.492438000	-0.000248000
C	3.226637000	-2.569938000	-0.000178000
C	2.601173000	-1.300325000	-0.000085000
H	-4.678385000	-1.714074000	-0.000117000

H	-3.226501000	-3.972927000	-0.000409000
H	-4.678638000	1.713381000	0.000077000
H	-0.372997000	-4.396912000	-0.000373000
H	-0.373642000	4.396853000	0.000347000
H	-3.227089000	3.972449000	0.000441000
H	2.292358000	4.568032000	0.000316000
H	4.288238000	2.747844000	0.000106000
H	2.293035000	-4.567699000	-0.000323000
H	4.288643000	-2.747210000	-0.000181000
Ni	-0.006682000	-0.000002000	0.000009000
C	3.352569000	0.000246000	0.000020000
F	4.200064000	0.000232000	1.092618000
F	4.200130000	0.000375000	-1.092523000

13. Ni[10-$(Me_3Si)_2$-isoCor] B3LYP/def2-TZVP

C	-3.913007000	-1.791787000	0.085623000
C	-2.985032000	-0.714715000	0.029595000
C	-3.164844000	-2.950911000	0.145926000
C	-2.985080000	0.713855000	-0.041147000
N	-1.731433000	-1.210202000	0.054274000
C	-1.787215000	-2.566707000	0.125382000
C	-3.913117000	1.790748000	-0.099550000
C	-0.607499000	-3.301477000	0.164704000
N	-1.731518000	1.209586000	-0.062581000
C	-1.787380000	2.566082000	-0.133799000
C	-0.607710000	3.301081000	-0.170078000
C	-3.165026000	2.950020000	-0.157893000
C	0.667480000	2.747425000	-0.135136000
N	0.979470000	1.389742000	-0.058771000
C	1.882395000	3.484820000	-0.168690000
C	2.906465000	2.581852000	-0.110615000
C	2.322890000	1.273822000	-0.041732000
C	0.667669000	-2.747579000	0.132987000
N	0.979586000	-1.389834000	0.057403000
C	1.882641000	-3.484743000	0.169492000
C	2.906678000	-2.581581000	0.113888000
C	2.323019000	-1.273662000	0.043614000
H	-4.987804000	-1.709125000	0.081778000
H	-3.534640000	-3.962875000	0.198686000
H	-4.987904000	1.707879000	-0.098488000
H	-0.669528000	-4.380984000	0.224228000
H	-0.669792000	4.380576000	-0.229752000
H	-3.534880000	3.961913000	-0.211589000
H	1.954010000	4.559844000	-0.225397000
H	3.955958000	2.804573000	-0.104404000
H	1.954320000	-4.559754000	0.226354000
H	3.956228000	-2.804094000	0.110155000
Ni	-0.306124000	-0.000170000	-0.002321000
C	3.094123000	0.000148000	0.001839000

Si	4.140545000	-0.020588000	-1.685759000
Si	4.136856000	0.021024000	1.691710000
C	5.529156000	1.257286000	-1.725832000
H	6.115828000	1.071119000	-2.629966000
H	5.175526000	2.285588000	-1.775853000
H	6.212357000	1.167636000	-0.879212000
C	4.986873000	1.660285000	2.074489000
H	5.562880000	1.508604000	2.992447000
H	5.688504000	1.983011000	1.304302000
H	4.281245000	2.469518000	2.256334000
C	5.525684000	-1.256531000	1.734740000
H	6.110581000	-1.070034000	2.639956000
H	5.172173000	-2.284895000	1.784326000
H	6.210495000	-1.166950000	0.889416000
C	2.910891000	-0.318848000	3.073359000
H	2.091669000	0.402981000	3.063317000
H	2.477322000	-1.317193000	3.004021000
H	3.414339000	-0.236276000	4.040366000
C	4.991806000	-1.659656000	-2.066587000
H	5.569640000	-1.507911000	-2.983387000
H	5.691966000	-1.982082000	-1.294938000
H	4.286786000	-2.469118000	-2.249772000
C	2.917537000	0.318882000	-3.070126000
H	2.098573000	-0.403261000	-3.061961000
H	2.483428000	1.317057000	-3.001722000
H	3.423188000	0.236543000	-4.036004000

14. Ni[10-Si(Me)$_2$-isoCor] B3LYP/def2-TZVP

C	-4.145215000	-1.763121000	-0.000127000
C	-3.188581000	-0.714947000	-0.000066000
C	-3.424238000	-2.939493000	-0.000262000
C	-3.188581000	0.714947000	0.000061000
N	-1.943439000	-1.222615000	-0.000116000
C	-2.042769000	-2.584536000	-0.000235000
C	-4.145216000	1.763121000	0.000234000
C	-0.901945000	-3.366920000	-0.000308000
N	-1.943439000	1.222615000	0.000093000
C	-2.042769000	2.584536000	0.000209000
C	-0.901945000	3.366920000	0.000285000
C	-3.424238000	2.939493000	0.000119000
C	0.393698000	2.869245000	0.000239000
N	0.772384000	1.521510000	0.000126000
C	1.558019000	3.682443000	0.000248000
C	2.635634000	2.835506000	0.000240000
C	2.121562000	1.508079000	0.000132000
C	0.393698000	-2.869245000	-0.000262000
N	0.772384000	-1.521510000	-0.000140000
C	1.558019000	-3.682443000	-0.000319000
C	2.635634000	-2.835506000	-0.000242000

C	2.121562000	-1.508079000	-0.000124000
H	-5.216848000	-1.648890000	-0.000090000
H	-3.811730000	-3.946124000	-0.000350000
H	-5.216849000	1.648890000	0.000303000
H	-1.014689000	-4.444131000	-0.000401000
H	-1.014689000	4.444131000	0.000333000
H	-3.811730000	3.946124000	0.000113000
H	1.556121000	4.761504000	0.000298000
H	3.680546000	3.104029000	0.000273000
H	1.556121000	-4.761504000	-0.000410000
H	3.680546000	-3.104029000	-0.000258000
Ni	-0.488126000	0.000000000	-0.000013000
Si	3.210116000	0.000000000	0.000037000
C	4.294283000	-0.000134000	1.537894000
H	4.937687000	-0.882673000	1.561029000
H	4.937707000	0.882387000	1.561170000
H	3.688377000	-0.000199000	2.445406000
C	4.294382000	0.000134000	-1.537749000
H	4.937800000	0.882664000	-1.560834000
H	4.937797000	-0.882395000	-1.560991000
H	3.688536000	0.000215000	-2.445301000

15. Ni[iso-10Me$_2$-5,15Ph$_2$C] OLYP/STO-TZP

Ni	0.000000000	0.000000000	-0.054355000
N	-1.385519000	0.280019000	1.217632000
N	1.385519000	-0.280019000	1.217632000
N	1.197235000	-0.218895000	-1.469967000
N	-1.197235000	0.218895000	-1.469967000
C	-2.735995000	0.543929000	0.922417000
C	-3.446461000	0.652256000	2.155371000
C	-2.538394000	0.468144000	3.169767000
C	-1.268326000	0.238817000	2.560250000
C	0.000000000	0.000000000	3.349035000
C	1.268326000	-0.238817000	2.560250000
C	2.538394000	-0.468144000	3.169767000
C	3.446461000	-0.652256000	2.155371000
C	2.735995000	-0.543929000	0.922417000
C	3.296916000	-0.653756000	-0.364493000
C	2.539544000	-0.489988000	-1.537368000
C	2.899686000	-0.583439000	-2.923072000
C	1.753168000	-0.354748000	-3.665876000
C	0.703914000	-0.133662000	-2.730952000
C	-0.703914000	0.133662000	-2.730952000
C	-1.753168000	0.354748000	-3.665876000
C	-2.899686000	0.583439000	-2.923072000
C	-2.539544000	0.489988000	-1.537368000
C	-3.296916000	0.653756000	-0.364493000
C	-4.751587000	0.961396000	-0.499025000
C	-5.650337000	-0.023241000	-0.935738000

C	-7.006759000	0.268253000	-1.083008000
C	-7.484044000	1.550709000	-0.807427000
C	-6.596085000	2.539049000	-0.379027000
C	-5.241312000	2.246085000	-0.221429000
C	4.751587000	-0.961396000	-0.499025000
C	5.241312000	-2.246085000	-0.221429000
C	6.596085000	-2.539049000	-0.379027000
C	7.484044000	-1.550709000	-0.807427000
C	7.006759000	-0.268253000	-1.083008000
C	5.650337000	0.023241000	-0.935738000
H	-5.284112000	-1.023759000	-1.152190000
H	-7.691416000	-0.510043000	-1.414690000
H	-8.541180000	1.778850000	-0.927088000
H	-6.957372000	3.543647000	-0.167928000
H	-4.553153000	3.021372000	0.106426000
H	5.284112000	1.023759000	-1.152190000
H	7.691416000	0.510043000	-1.414690000
H	8.541180000	-1.778850000	-0.927088000
H	6.957372000	-3.543647000	-0.167928000
H	4.553153000	-3.021372000	0.106426000
H	3.889348000	-0.796581000	-3.304177000
H	1.662860000	-0.348782000	-4.745543000
H	-1.662860000	0.348782000	-4.745543000
H	-3.889348000	0.796581000	-3.304177000
H	-4.507118000	0.836475000	2.254618000
H	-2.748941000	0.486894000	4.230381000
H	2.748941000	-0.486894000	4.230381000
H	4.507118000	-0.836475000	2.254618000
C	0.229646000	1.253917000	4.254384000
H	0.377972000	2.147084000	3.640844000
H	-0.630673000	1.424464000	4.904683000
H	1.110487000	1.122394000	4.885994000
C	-0.229646000	-1.253917000	4.254384000
H	0.630673000	-1.424464000	4.904683000
H	-1.110487000	-1.122394000	4.885994000
H	-0.377972000	-2.147084000	3.640844000

16. Ni[iso-10Me,10H-5,15Ph$_2$C] B3LYP/def2-TZVP

C	-3.591708000	-1.795092000	0.066862000
C	-2.662987000	-0.719349000	0.081668000
C	-2.843607000	-2.957115000	0.021252000
C	-2.663092000	0.718935000	0.081781000
N	-1.411878000	-1.214442000	0.053862000
C	-1.467392000	-2.574699000	0.009080000
C	-3.591968000	1.794546000	0.067154000
C	-0.290038000	-3.314375000	-0.061606000
N	-1.412055000	1.214215000	0.054052000
C	-1.467766000	2.574470000	0.009486000
C	-0.290518000	3.314329000	-0.061071000

C	-2.844036000	2.956684000	0.021704000
C	0.987318000	2.768122000	-0.076565000
N	1.286945000	1.404244000	-0.006962000
C	2.212517000	3.489355000	-0.146071000
C	3.229111000	2.568756000	-0.109331000
C	2.616124000	1.285789000	-0.019257000
C	0.987720000	-2.767980000	-0.077017000
N	1.287148000	-1.404071000	-0.007183000
C	2.213022000	-3.489023000	-0.146658000
C	3.229483000	-2.568281000	-0.109778000
C	2.616310000	-1.285419000	-0.019476000
H	-4.666214000	-1.712105000	0.084304000
H	-3.215185000	-3.969470000	-0.004259000
H	-4.666462000	1.711401000	0.084590000
H	-0.362034000	-4.394085000	-0.110779000
H	-0.362671000	4.394036000	-0.110072000
H	-3.215762000	3.968989000	-0.003658000
H	2.295991000	4.562792000	-0.214597000
H	4.289851000	2.759191000	-0.142840000
H	2.296652000	-4.562436000	-0.215365000
H	4.290250000	-2.758555000	-0.143334000
Ni	0.007479000	-0.000009000	0.034439000
C	3.376408000	0.000230000	0.091340000
H	4.102261000	0.000353000	-0.732203000
C	4.210795000	0.000178000	1.404651000
H	4.845449000	-0.883464000	1.455497000
H	4.845322000	0.883901000	1.455648000
H	3.547798000	0.000056000	2.270259000

17. Ni[iso-10Me$_2$-5,15Ph$_2$C] B3LYP/def2-TZVP

C	-3.607469000	-1.794767000	-0.000178000
C	-2.678958000	-0.719362000	-0.000064000
C	-2.858667000	-2.957580000	-0.000224000
C	-2.679061000	0.718957000	0.000076000
N	-1.427496000	-1.213434000	-0.000109000
C	-1.483339000	-2.574200000	-0.000234000
C	-3.607727000	1.794230000	0.000216000
C	-0.303475000	-3.311607000	-0.000305000
N	-1.427670000	1.213209000	0.000114000
C	-1.483710000	2.573968000	0.000231000
C	-0.303952000	3.311544000	0.000298000
C	-2.859093000	2.957150000	0.000178000
C	0.973119000	2.764432000	0.000243000
N	1.280373000	1.399387000	0.000133000
C	2.192856000	3.495013000	0.000249000
C	3.213631000	2.580766000	0.000204000
C	2.611468000	1.286839000	0.000107000
C	0.973517000	-2.764312000	-0.000250000
N	1.280574000	-1.399222000	-0.000130000

C	2.193360000	-3.494717000	-0.000301000
C	3.214003000	-2.580323000	-0.000187000
C	2.611652000	-1.286484000	-0.000099000
H	-4.682073000	-1.711320000	-0.000183000
H	-3.229381000	-3.970559000	-0.000284000
H	-4.682319000	1.710627000	0.000252000
H	-0.370829000	-4.392758000	-0.000392000
H	-0.371460000	4.392685000	0.000358000
H	-3.229953000	3.970076000	0.000201000
H	2.270718000	4.571045000	0.000308000
H	4.270368000	2.789953000	0.000217000
H	2.271377000	-4.570737000	-0.000396000
H	4.270771000	-2.789355000	-0.000177000
Ni	-0.003914000	-0.000010000	0.000001000
C	3.401958000	0.000233000	0.000027000
C	4.309611000	0.000191000	1.264706000
H	4.948399000	-0.882059000	1.277420000
H	4.948318000	0.882497000	1.277536000
H	3.705050000	0.000104000	2.171990000
C	4.309696000	0.000390000	-1.264589000
H	4.948398000	0.882702000	-1.277243000
H	4.948492000	-0.881852000	-1.277393000
H	3.705197000	0.000436000	-2.171914000

18. Ni[10-isoCor] B3LYP/def2-TZVP

C	3.217052000	2.801619000	0.000169000
C	1.856897000	2.388028000	0.000054000
C	3.977128000	1.647129000	0.000226000
C	0.543218000	2.969866000	-0.000081000
N	1.804046000	1.042613000	0.000098000
C	3.070663000	0.542381000	0.000233000
C	-0.064548000	4.255070000	-0.000200000
C	3.275401000	-0.834979000	0.000317000
N	-0.417591000	2.026624000	-0.000120000
C	-1.639225000	2.628428000	-0.000237000
C	-2.796769000	1.854415000	-0.000303000
C	-1.430268000	4.042111000	-0.000215000
C	-2.816906000	0.464834000	-0.000255000
N	-1.687523000	-0.358675000	-0.000173000
C	-3.973413000	-0.366453000	-0.000230000
C	-3.539704000	-1.668576000	-0.000142000
C	-2.117029000	-1.621364000	-0.000099000
C	2.259902000	-1.783699000	0.000271000
N	0.891099000	-1.500721000	0.000164000
C	2.421448000	-3.198789000	0.000294000
C	1.165704000	-3.752610000	0.000151000
C	0.244626000	-2.667292000	0.000093000
H	3.575714000	3.818067000	0.000173000
H	5.053517000	1.576711000	0.000295000

H	0.447146000	5.203737000	-0.000218000
H	4.293536000	-1.204630000	0.000404000
H	-3.754735000	2.359927000	-0.000362000
H	-2.205760000	4.791902000	-0.000263000
H	-4.991600000	-0.009803000	-0.000282000
H	-4.138075000	-2.565905000	-0.000110000
H	3.369784000	-3.713138000	0.000388000
H	0.903251000	-4.798731000	0.000112000
Ni	0.118865000	0.237814000	-0.000008000
C	-1.236470000	-2.822326000	-0.000029000
H	-1.510359000	-3.441009000	-0.864842000
H	-1.510523000	-3.441014000	0.864727000
